# Spatial and Temporal Variations in Richness, Diversity and Abundance of Floral Visitors of Curry Plants (*Bergera koenigii* L.): Insights on Plant-Pollinator Interactions

**DOI:** 10.3390/insects15020083

**Published:** 2024-01-24

**Authors:** Ujjwal Layek, Anirban Deep Das, Uday Das, Prakash Karmakar

**Affiliations:** 1 Department of Botany, Rampurhat College, Rampurhat 731224, India; layekujjwal@yahoo.co.in (U.L.); 20anirbandeepdas@gmail.com (A.D.D.); udaydas.vb@gmail.com (U.D.); 2 Department of Botany & Forestry, Vidyasagar University, Midnapore 721102, India

**Keywords:** butterfly, flower visitor, honeybee, *Murraya koenigii*, NDVI, solitary bee, stingless bee, spatiotemporal, visitor activity

## Abstract

**Simple Summary:**

Flower-visitor communities and plant-pollinator interactions are species- and region-specific and may vary spatially and temporally. We studied flower visitor interactions with curry plants, considering both daily fluctuations and differences across zones characterized by varying vegetation densities (i.e., dense, medium-density, and low-density vegetation). The visitors’ richness, diversity, and abundance were higher in the area with dense vegetation. Specifically, between 10.00 h and 14.00 h, these parameters reached their peak, contrasting with lower activity observed during early mornings and late afternoons. For most visitors, the flower handling time was lower, and the visitation rate was higher in dense vegetation areas (at 10.00–14.00 h) than in medium- and low-density vegetation areas. The proportions of foraging categories varied over time, with higher ratios of mixed foragers observed in the early morning. Bee species, such as *Apis cerana*, *Apis dorsata*, *Halictus acrocephalus*, *Nomia iridescens*, and *Tetragonula iridipennis*, and butterfly species, such as *Appias libythea*, were the most effective pollinators of curry plants. The effectiveness of pollinators also varied spatially and remained region-specific. In conclusion, flower-visitor communities and plant-pollinator interactions varied spatially and temporally.

**Abstract:**

The reproductive success of flowering plants relates to flower-visitor communities and plant-pollinator interactions. These traits are species- and region-specific and vary across regions, pollinator groups, and plant species. However, little literature exists on the spatiotemporal variation in visitor activity, especially in India. Here, we aimed to depict the spatial and temporal variation in visitor activity on the curry plants (*Bergera koenigii*). Data were collected at different daytime slots from three vegetation zones (confirmed by field surveys and normalized difference vegetation index values in remote sensing)—dense, medium-density, and low-density vegetation in West Bengal, India. The visitors’ richness, diversity, and abundance were higher in the area with dense vegetation. Considering daytime patterns, higher values for these parameters were obtained during 10.00–14.00 h. For most visitors, the flower handling time was shorter, and the visitation rate was higher in dense vegetation areas (at 10.00–14.00 h) than in medium- and low-density vegetation areas. The proportions of different foraging categories varied over time. Vital pollinators were *Apis cerana*, *Apis dorsata*, *Appias libythea*, *Halictus acrocephalus*, *Nomia iridescens*, and *Tetragonula iridipennis*. However, the effectiveness of pollinators remained region-specific. Therefore, it can be concluded that floral visitors’ richness, diversity, abundance, and plant-visitor interactions varied spatially with their surrounding vegetation types and also changed daytime-wise.

## 1. Introduction

Most floral visitors mutually associate with flowering plants, collecting floral rewards and providing pollination services to many wild and crop plants [1,2,3]. The reproductive fitness of plants is highly dependent on ecosystem services and plant-pollinator interactions. The study of pre-pollination interaction has remained at the forefront in the fields of pollination biology and community ecology [4,5,6].

Most plants have a wide distribution range and different eco-climatic conditions. Floral visitors’ composition and their interactions with particular plant species can vary from region to region. These changes in flower visitor species composition can have strong (or negligible) effects on plant reproductive success. The spatial variation in floral visitor activity and interactions shapes a broad pattern of pollinator diversity [7,8]. The diverse pollinator community can buffer against eco-climatic variability [9]. A diverse community of pollinators can also increase plant reproductive success, as each insect species has different functional traits and pollination strategies, especially if species respond differentially to environmental variation or disturbance [10,11].

Anthropogenic habitat alteration and habitat degradation caused by the conversion of semi-natural to human-dominated habitats are the primary threats to pollinator diversity [12,13]. Growing urbanisation, as a global driver of land use change, is thought to have a negative impact on pollinator composition [14,15]. The effect of land use change and local habitat on plants and insect pollinators is also likely to influence their interactions, thereby affecting the network architecture [16,17]. The interaction networks and species composition could be important in promoting community stability and functionality [18,19]. Despite the growing sphere of pollination biology research in plant systems, little is known about the impacts of local habitats on floral visitors’ composition and interactions that may vary spatially and temporally.

In this study, we aimed to quantify floral visitors’ composition of curry plants [*Bergera koenigii* (L.), syn. *Murraya koenigii* (L.) Sprengel] (as only little data is available and those came from outside the state) and intended to uncover the spatio-temporal variation in floral visitors and plant-pollinator interactions. In this context, the present study addresses the following questions: (1) What are the floral visitors of the plant species? (2) Does a floral visitor’s richness, diversity, and abundance vary spatio-temporally? (3) Are the plant-pollinator interactions changing spatio-temporally in accordance with vegetation types?

## 2. Materials and Methods

### 2.1. Plant Species

The present works were conducted on curry plants [*Bergera koenigii*, syn. *Murraya Koenigii*; family: Rutaceae], during 2019–2021. It is commonly known as a curry tree or curry bush. The plant is native to the Indian subcontinent. Fresh leaves are an indispensable part of Indian traditional medicines. The curry leaves are frequently used to flavour different food items. In spring, the plant produces a huge number of small white flowers and can provide floral resources to various insect species. However, there is insufficient data about its floral visitors and their interactions with the plant species.

### 2.2. Study Sites and Vegetation Patterns

We carried out the present works in three zones: (i) Bolpur of Birbhum district, (ii) Jenadihi of Bankura district, and (iii) Rangamati of Midnapore town of Paschim Medinipur district, West Bengal, India. To characterize the vegetation types (i.e., dense vegetation, medium-density vegetation, and low-density vegetation), we surveyed 1.5 km around the selected plants. We counted the number of trees and shrubs per 20 m × 20 m quadrat (20 quadrats per zone). We also applied remote sensing technology to map vegetation covers over the land areas. We estimated the normalized difference vegetation index (NDVI) using QGIS.

### 2.3. Data Collection about Floral Visitors

We observed the visitors at six time-slots (i.e., 6.00–8.00 h, 8.00–10.00 h, 10.00–12.00 h, 12.00–14.00 h, 14.00–16.00 h, and 16.00–18.00 h) during peak flowering time (i.e., mid-February–mid-April). A direct observation method was followed to encounter floral visitors. Each survey (i.e., plant-based sampling) was continued for 5 min on an inflorescence. The visitors encountered were identified in the field or captured (with the help of an insect net) for later identification. We estimated the availability or abundance (i.e., the average number of individuals of a species/inflorescence/5 min) of the flower-visiting species daytime-wise and zone-wise. We also calculated the zone-wise relative abundance (RA) of each insect species as follows:

$$RA\left(\%\right)=\frac{ni}{N}\times 100$$

where n*i* is the number of encountered individuals of the insect species *i*, and N is the total number of encountered individuals for all the flower visitors of the plant species.

We recorded the flower visitation rate (VR) or foraging rate as the number of flowers visited in a 1 min duration. We recorded data 10 times per timeslot per zone for an insect species. The flower handling time (i.e., the amount of time spent per visit on a flower) was also recorded (*n* = 10 × 6 observations for a flower-visiting species per zone).

We documented the resources collected by the floral visitors and estimated the proportion of each of the three foraging-task allocation categories─ (i) specialized nectar foragers: collect only nectar on a single bout; (ii) specialized pollen foragers: collect only pollen grains on a single bout; and (iii) mixed foragers: collect both nectar and pollen grains on a single bout. We closely observed flower-visiting individuals, whether an individual collects nectar, pollen, or both (nectar collection: the forager sucks nectar from flowers; pollen collection: brush their body with legs and may have stacked pollen on scopae, corbiculae, or abdomen). In this way, we conducted 30 sampling observations (5 × 6 samples; 6-time slots) for each insect species per vegetation zone (one sample comprises ten individuals) and compared zone-wise and daytime-wise.

We estimated the pollen-carrying value (PCV) of floral visitors according to the method of Layek et al. [20] by summing two components: (i) PCV 1 (based on the average number of pollen grains attached to a visitor body surface, omitting stack pollen loads on abdomen, corbiculae, or scopae; value ranged from 0 to 5) and (ii) PCV 2 (based on the average number of pollen grains found in stack pollen loads on abdomen, corbiculae, or scopae; value ranged from 0 to 3). We entrapped the flower-visiting species with an insect collecting net to estimate the number of pollen grains carried by an insect species. We carefully observed the captured insects (in the case of bees) to see whether there was stacked pollen or not. We kept the arrested individuals (without stacked pollen loads) in a vial (one individual per vial) with 1.0–5.0 mL of sucrose solution. After shaking, we removed the insects from the vial and left the solution. Then, we took 10 µL of the solution (using a micropipette) on a slide and counted the number of pollen grains under microscopic study. We calculated the total number of pollen grains adhering to the insect body by multiplying with the proper factors (e.g., by 100 if the initial solution volume was 1 mL). In the case of the stacked pollen load count, we scraped the loads and took them in a vial. Then, we added 1 mL of sucrose solution, shook, and counted the pollen by following the method mentioned above. Then, we calculated the approximate pollination value (APV) for the flower-visiting species by multiplying the numerical values of three individual parameters (in order to address their role as pollinators of the plant species) as follows (Layek et al. [20]):

APV=RA×VR×PCV

### 2.4. Data Analysis

The richness of the flower-visiting community was calculated using Margalef’s index, D [D = (S − 1)/ln N; S is the number of species and N is the total number of individuals]. The diversity of floral visitors was estimated using Shannon–Wiener diversity index (*H*′) as follows:

$$H^{\prime }=-\sum_{i}^{n}\left(pi.\ln pi\right)$$

where *H*′ is the diversity index and pi is the proportion of each visitor species found within the sample. This proportion is calculated as ni/N, where ni is the number of individuals encountered for *i* species and N is the total number of individuals encountered in the sample. The natural logarithm is denoted by *ln*. Here, one observation (5 min on an inflorescence) was considered a sample for estimating the species richness and diversity.

The data was analysed descriptively to get the mean and standard deviation. We used the ‘Shapiro–Wilk’ test to determine if the data were normally distributed or not. We used a parametric test called ‘One-way ANOVA’ on normal distributed data (e.g., flower visitation rate and flower handling time). We used Duncan’s multiple range test for post hoc comparisons if the derived *p*-value was significant. In the case of a non-normal distribution (e.g., visitor’s abundance, diversity, and richness), we performed a non-parametric independent-samples Kruskal–Wallis test. If the obtained *p*-value was significant, we conducted pairwise comparisons of interventions using Dunn’s post hoc test. Here, *p* ≤ 0.05 was judged statistically significant. SPSS (ver. 25.0) statistics packages were used for the statistical analyses.

## 3. Results

### 3.1. Vegetation Patterns in the Three Selected Zones

**Figure 1:**
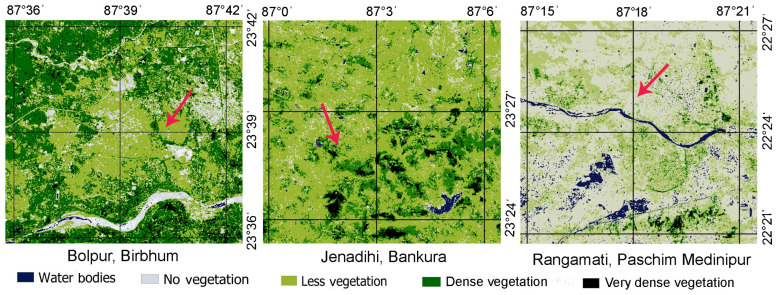
NDVI maps of the three study sites (i.e., Bolpur, Jenadihi, and Rangamati) in West Bengal have different vegetation patterns. The arrows indicate sampling areas.

The surrounding vegetation of selected curry plants at Bolpur (in Birbhum district) consists of a few trees, shrubs and weeds. At Jenadihi (in Bankura district), the surrounding vegetation comprises many trees, shrubs, and weeds. A few crop fields were also there. Whereas, at Rangamati (in Paschim Medinipur district), only a few trees are noticed. The number of trees and shrubs per quadrat (20 m × 20 m) was higher in Jenadihi (trees: 7.95 ± 4.39; shrubs: 8.80 ± 5.38), followed by Bolpur (trees: 4.60 ± 2.58; shrubs: 8.60 ± 4.07) and Rangamati (trees: 3.15 ± 3.15; shrubs: 4.80 ± 3.02). From NDVI data, it was revealed that dense vegetation areas are at Jenadihi, medium-density vegetation areas at Bolpur, and low-density vegetation areas in Rangamati (Figure 1).

### 3.2. Floral Visitors

**Figure 2:**
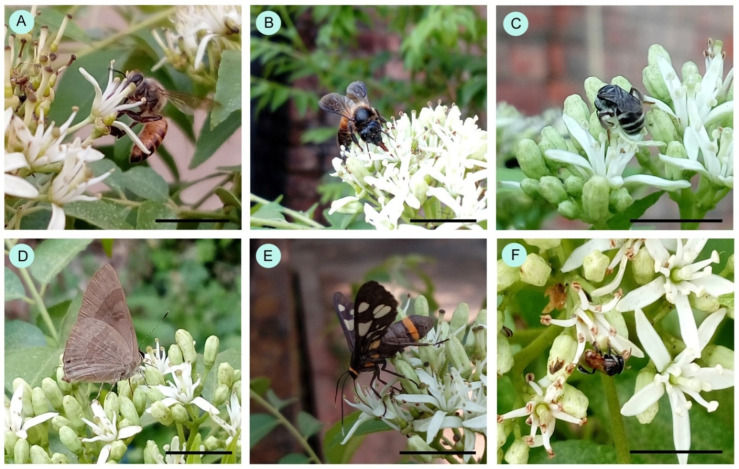
Some floral visitors to the curry plants. (**A**) *Apis cerana*, (**B**) *Apis dorsata*, (**C**) *Halictus acrocephalus*, (**D**) *Rapala varuna*, (**E**) *Syntomoides imaon*, and (**F**) *Tetragonula iridipennis*. Scale bars are 10 mm.

A total of 45 insect species were documented as floral visitors to curry in West Bengal, India (Table 1, Figure 2). Zone-wise, there were 44 insect species from the dense vegetation area (at Jenadihi), 33 from the medium-density vegetation area (at Bolpur), and 29 from the low-density vegetation area at Rangamati. The most represented insect orders were Hymenoptera (13 species) and Lepidoptera (30 species). Among the hymenopteran members, most belong to the Apidae (8 species) and Halictidae (4 species). Most butterflies belong to the insect families Lycaenidae (8 species), Nymphalidae (8 species), and Pieridae (6 species).

Overall, species richness was higher in dense vegetation areas (Margalef’s index, D = 6.43) than in medium- and low-density areas (D values were 4.88 and 4.37, respectively). Sample-wise visitor’s richness also differed among the three sites (Kruskal-Wallis *χ*^2^ = 26.89, *p* < 0.001, *df* = 2). Comparatively higher species richness was obtained for dense vegetation (D = 0.94 ± 0.72) than the medium- and low-density vegetation areas (D = 0.77 ± 0.64 and 0.60 ± 0.64 for medium and low-density vegetation areas, respectively). Flower-visiting species richness also varied daytime-wise (Bolpur: Kruskal-Wallis *χ*^2^ = 38.52, *p* < 0.001, *df* = 5; Jenadihi: Kruskal-Wallis *χ*^2^ = 69.99, *p* < 0.001, *df* = 5; Rangamati: Kruskal-Wallis *χ*^2^ = 35.84, *p* < 0.001, *df* = 5). Comparatively higher species richness was recorded during 10.00–14.00 h than in the early morning and late afternoon (Table 2).

The diversity of insect visitors differed among these three vegetation areas (Kruskal-Wallis *χ*^2^ = 28.80, *p* < 0.001, *df* = 2). Visitor’s diversity remained lower in low-density vegetation areas (Shannon–Weiner index *H*’ = 0.53 ± 0.44) than in dense and medium-density vegetation areas (*H*’ = 0.74 ± 0.48 for dense vegetation areas and *H*’ = 0.67 ± 0.47 for medium-density vegetation areas). Irrespective of the vegetation zones, the daytime-wise difference was also noticed (Bolpur: Kruskal-Wallis *χ*^2^ = 109.53, *p* < 0.001, *df* = 5; Jenadihi: Kruskal-Wallis *χ*^2^ = 117.70, *p* < 0.001, *df* = 5; Rangamati: Kruskal-Wallis *χ*^2^ = 79.37, *p* < 0.001, *df* = 5). The higher diversity was obtained in 10.00–14.00 h and comparatively lower in the early morning and late afternoon (Table 3).

**Figure 3:**
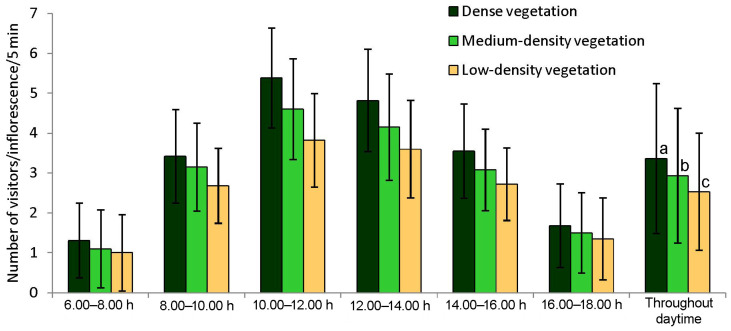
The abundance of floral visitors on curry plants (*Bergera koenigii*), considering daytime and zone-wise. Data indicate the mean ± standard deviation. Different letters indicate significant differences (Dunn’s post hoc test at 5%).

The abundance of insect visitors also differed among the three zones (Kruskal-Wallis *χ*^2^ = 23.65, *p* < 0.001, *df* = 2). A higher abundance of floral visitors was recorded for the dense vegetation area (3.36 ± 1.88 visitors/inflorescence/5 min) than the medium- and low-density vegetation areas (2.93 ± 1.69 visitors/inflorescence/5 min and 2.53 ± 1.47 visitors/inflorescence/5 min) (Figure 3). Daytime-wise visitor’s abundance also fluctuated (dense vegetation: Kruskal-Wallis *χ*^2^ = 155.58, *p* < 0.001, *df* = 5; medium-density vegetation: Kruskal-Wallis *χ*^2^ = 137.99, *p* < 0.001, *df* = 5; low-density vegetation: Kruskal-Wallis *χ*^2^ = 122.62, *p* < 0.001, *df* = 5). Visitor’s abundance was high at 10.00–12.00 h and low in the early morning and late afternoon. The relative abundance of flower-visiting species varied from zone to zone (Supplementary Table S1). In the dense vegetation area of Jenadihi village, the most abundant floral visitors were *Appias libythea* (RA = 14.64%), *Halictus acrocephalus* (RA = 9.06%), and *Nomia iridescens* (RA = 17.49%). In the medium-density vegetation area of Bolpur, *Halictus acrocephalus* (RA = 17.78%), *Nomia iridescens* (RA = 8.82%), and *Tetragonula iridipennis* (RA = 15.08%) were dominant. In the low-density vegetation of Rangamati, abundant visitors were *Apis cerana* (RA = 8.73%), *Halictus acrocephalus* (RA = 16.97%), and *Tetragonula iridipennis* (RA = 20.43%).

Flower visitation rates were comparatively higher in honeybees and solitary bees than the lepidopteran members (except the moth *Syntomoides imaon*) and stingless bees (Table 4). The visitation rate of most insect species varied zone-wise (e.g., *Apis cerana*: *F*_2,177_ = 9.52, *p* < 0.001; *Apis dorsata*: *F*_2,177_ = 21.23, *p* < 0.001; *Appias libythea*: *F*_2,177_ = 14.75, *p* < 0.001; *Halictus acrocephalus*: *F*_2,177_ = 36.26, *p* < 0.001; *Nomia iridescens*: *F*_1,118_ = 9.91, *p* < 0.01; *Tetragonula iridipennis*: *F*_2,177_ = 19.47, *p* < 0.001). Greater visitation rates were recorded in dense vegetation areas than in medium- and low-density vegetation areas (e.g., *Apis cerana*: 11.05 ± 2.49, 9.90 ± 2.40 and 9.12 ± 2.43 flowers/min; *Apis dorsata*: 19.53 ± 3.68, 16.10 ± 4.43, and 14.98 ± 3.81 flowers/min; *Appias libythea*: 5.62 ± 1.75, 4.55 ± 1.59 and 4.07 ± 1.45 flowers/min; *Halictus acrocephalus*: 8.92 ± 2.49, 6.82 ± 2.27, and 5.35 ± 2.15 flowers/min; *Nomia iridescens*: 11.92 ± 2.78 and 10.28 ± 2.90 flowers/min; *Tetragonula iridipennis*: 2.80 ± 1.06, 2.20 ± 0.96 and 2.00 ± 0.95 flowers/min. The values were given sequentially as dense, medium-, and low-density vegetation zones, respectively). The visitation rate also varied daytime-wise (e.g., *Apis cerana*: *F*_5,174_ = 16.34, *p* < 0.001; *Apis dorsata*: *F*_5,174_ = 19.37, *p* < 0.001; *Appias libythea*: *F*_5,174_ = 13.55, *p* < 0.001; *Halictus acrocephalus*: *F*_5,174_ = 14.15, *p* < 0.001; *Nomia iridescens*: *F*_5,114_ = 8.31, *p* < 0.001; *Tetragonula iridipennis*: *F*_5,174_ = 5.39, *p* < 0.001). The visitation rates were higher during 10.00–14.00 h and lower in the early morning and late afternoon (Supplementary Table S2).

The flower handling times were very low for *Amegilla zonata*, *Apis dorsata*, and *Syntomoides imaon*. Comparatively higher handling times were recorded for butterflies and stingless bees (Table 4). The flower handling times of most insect species varied zone-wise (e.g., *Apis cerana*: *F*_2,177_ = 4.11, *p* < 0.05; *Appias libythea*: *F*_2,177_ = 16.04, *p* < 0.001; *Halictus acrocephalus*: *F*_2,177_ = 16.19, *p* < 0.001; *Nomia iridescens*: *F*_1,118_ = 4.58, *p* < 0.05; *Tetragonula iridipennis*: *F*_2,177_ = 19.13, *p* < 0.001). The lower flower handling times were recorded in dense vegetation areas than in medium- and low-density vegetation areas (e.g., *Apis cerana*: 3.66 ± 1.15 s, 4.03 ± 1.24 s and 4.32 ± 1.41 s; *Appias libythea*: 4.83 ± 2.10 s, 6.02 ± 2.18 s and 7.07 ± 2.23 s; *Halictus acrocephalus*: 6.22 ± 2.50 s, 7.85 ± 3.33 s and 9.55 ± 3.67 s; *Nomia iridescens*: 5.44 ± 3.09 and 6.25 ± 3.69 flowers/min; *Tetragonula iridipennis*: 11.76 ± 3.71, 14.03 ± 4.04 and 16.34 ± 4.40 flowers/min. The values were given sequentially for dense, medium-, and low-density vegetation zones. Flower handling time also varied daytime-wise (*Apis cerana*: *F*_5,174_ = 18.62, *p* < 0.001; *Appias libythea*: *F*_5,174_ = 4.00, *p* < 0.001; *Halictus acrocephalus*: *F*_5,174_ = 5.95, *p* < 0.001; *Nomia iridescens*: *F*_5,114_ = 2.66, *p* < 0.05; *Tetragonula iridipennis*: *F*_5,174_ = 2.43, *p* < 0.05). The flower handling times were lower during 10.00–14.00 h and higher in the early morning (Supplementary Table S3).

Among the floral visitors, we recorded specialized nectar foragers and mixed foragers (individual foragers collecting both nectar and pollen grains). However, specialized pollen foragers were rare on curry flowers (only recorded for some individuals of stingless bees). Some flower-visiting insects (e.g., butterflies, cuckoo bees, flies, moths, and wasps) were exclusively nectar foragers (Supplementary Table S4). Most bee species (e.g., *Amegilla zonata*, *Apis* spp., *Halictus acrocephalus*, and *Tetragonula iridipennis*) collected both nectar and pollen grains. The proportion of each category varied insect species-wise. The ratio of mixed foragers (i.e., foragers who collect nectar and pollen grains) was comparatively higher in *Amegilla zonata* (41.11 ± 34.69%), *Halictus acrocephalus* (46.11 ± 38.44%), and *Tetragonula iridipennis* (42.22 ± 35.15%) than honeybees (*Apis cerana*: 33.67 ± 28.22%, *Apis dorsata*: 13.56 ± 13.35%). The proportion of nectar foragers and mixed foragers did not vary from zone to zone but significantly varied daytime-wise (e.g., mixed foragers: *Amegilla zonata*: Kruskal-Wallis *χ*^2^ = 85.00, *p* < 0.001, *df* = 5; *Halictus acrocephalus*: Kruskal-Wallis *χ*^2^ = 85.35, *p* < 0.001, *df* = 5; *Tetragonula iridipennis*: Kruskal-Wallis *χ*^2^ = 83.64, *p* < 0.001, *df* = 5). The proportion of mixed foragers was comparatively higher during the early morning, and mixed foragers were not found in the late afternoon (Supplementary Table S4).

The pollen-carrying values (PCV) were higher for blue-banded bees, honeybees, solitary bees, and stingless bees. The PCV was very low for butterflies (Table 5). Based on the approximate pollination value (APV), vital pollinators of curry plants in dense vegetation areas were *Apis dorsata*, *Halictus acrocephalus*, and *Nomia iridescens*; in medium-density vegetation areas, *Apis dorsata*, *Halictus acrocephalus*, *Nomia iridescens*, and *Tetragonula iridipennis*; and in low-density vegetation areas, *Apis cerana*, *Apis dorsata*, *Halictus acrocephalus*, and *Tetragonula iridipennis*.

## 4. Discussion

Many insect species (i.e., 45 species) belonging to different groups (e.g., butterflies, flies, honeybees, moths, solitary bees, stingless bees, and wasps) were recorded as floral visitors of curry plants (*Bergera koenigii*) from West Bengal, India. From outside the state, very few flower-visiting species were reported for curry flowers (Bhatnagar et al. [21]: five hymenopteran members and three lepidopteran members; Dhore [22]: five hymenopteran members and five lepidopteran members) compared with our present study (here, 13 species in Hymenoptera and 30 species in Lepidoptera were recorded). Concerning the diverse floral visitors, the plant species can be treated as magnetic plants for bees and butterflies, like other plants (e.g., *Chromolaena odorata*: Layek et al. [23]; *Foeniculum vulgare*: Layek et al. [20]) in West Bengal. For that, curry plants may play a vital role in insect conservation, which was narrated for the first time by us in the present study. Most flower-visiting insects belong to the orders Lepidoptera (mostly butterflies) and Hymenoptera. Flowers’ preference for butterflies may depend on their proboscis length, which correlates with the length of the flower corolla tube [24]. Butterfly species with a high wing load prefer to visit clustered or nectar-rich flowers. In contrast, their low wing loading limited their visits to solitary flowers with less nectar-rich [25]. Here, curry flowers (i.e., clustered and less nectar-rich) were visited by both high-wing and low-wing-loading species. This may be due to the rich species composition of butterflies within the study sites.

Floral visitors’ abundance, richness, and diversity varied zone-wise, with a higher value in dense vegetation than in medium- and low-density vegetation areas. Many researchers (e.g., Layek et al. [26]; Gilpin et al. [27]) reported a higher abundance and richness of floral visitors in the region with a greater area of native vegetation and higher floral richness. In dense vegetation zones, diverse insect species are well-suited due to the greater availability of floral resources and nesting habitats. In dense vegetation, diverse flora may support the sustenance of numerous insect species. These parameters also varied daytime-wise, with higher abundance, diversity, and richness at 10.00–14.00 h. The probable explanation is that the foraging activity may be high for most curry visitors at this time because of optimum weather conditions (including temperature, light, and humidity). The most abundant visitors on curry were *Apis cerana*, *Apis dorsata*, *Appias libythea*, *Halictus acrocephalus*, *Nomia iridescens*, and *Tetragonula iridipennis*. The relative abundance of the insect species is highly varied vegetation-wise. *Halictus acrocephalus* dominated in all three zones but varied in its proportions. *Nomia iridescens* dominates dense and medium-density vegetation zones. *Halictus acrocephalus* is probably capable of managing required foodstuffs and nesting habitats in versatile landscapes, including disturbed areas. Therefore, the populations of *Halictus acrocephalus* are less sensitive to altered land uses and vegetation. While populations of *Nomia iridescens* are more susceptible to vegetation degradation, it may be due to unavailable floral resources or nesting substrates. In low-density vegetation (i.e., highly disturbed areas), stingless bees and honeybees were dominant. The dominance of honeybees can be explained by the presence of a large number of colonies during the blooming period of curry. There may be an influence of managed colonies of *Apis cerana* on the Vidyasagar University campus (about 1 km from the studied plants). The higher abundance of stingless bees can be explained by the presence of perennial colonies of stingless bees near the selected plants and less competition and aggression from other floral visitors.

The interaction of visitors with curry plants also varied from zone to zone as well as daytime-wise. In most flower-visiting species, flower handling time was lower, and the visitation rate was higher in dense vegetation than in medium- and low-density vegetation areas. The higher completion rate (with a greater abundance of visitors) for resource collection and the aggression of different visitors in dense vegetation can reduce flower handling time and increase the visitation rate [28,29]. Considering daytime, flower handling times were higher in the early morning, and flower visitation rates were lower then. This may be related to the amount of floral resource availability and the foraging activity of the visitors.

The proportion of each resource-collecting task allocation category (i.e., nectar foragers, pollen foragers, and mixed foragers) varied species-wise and daytime-wise. Butterflies, moths, and wasps do not feed on pollen, so they do not actively collect pollen. The collection of only nectar resources by butterflies and wasps was also reported for the curry plants [22], as well as for other plants [23,30]. Some flower-visiting bees (e.g., *Sphecodes gibbus* and *Thyreus nitidulus*) are cleptoparasitic species that lay eggs in the nests of other bees and never collect pollen themselves. Thus, they would only collect nectar from flowers to support their flight. The supremacy of pollen foragers is not reported for curry flowers. The pollen-collecting behavior of visitors depends on complex parameters, including resource quality, quantity, accessibility, and availability [31,32,33]. Significant proportions of mixed foragers were recorded in some insects (e.g., *Amegilla zonata*, *Ceratina* spp., *Halictus acrocephalus*, and *Tetragonula iridipennis*). Mixed foraging behavior (collecting both nectar and pollen by an individual forager on a single bout) remains a more profitable foraging strategy considering flight costs (energy and time). However, profitability depends on species, sex, and life-history traits such as sociality, floral specialisation, life cycle, etc. The proportion of each category did not vary zone-wise for a particular insect species but varied daytime-wise. Comparatively higher percentages of mixed foragers were recorded in the early morning. In the early morning, there was greater pollen availability for the visitors. The resource-collecting task allocation depends on the availability and accessibility of floral resources and also on colony demands.

Most bees (excluding *Sphecodes gibbus* and *Thyreus nitidulus*) have greater pollen-carrying values (PCV) than butterflies, flies, moths, and wasps. Pollen-carrying values for an insect species depend on its morphometry, resource-collecting behavior, floral architecture, and pollen yield of the flower. The higher PCV of bees may be because they are more hairy and vigorously touch the flower’s anthers. Pollen collection behavior (as they have a significant proportion of mixed foragers) may also raise their pollen-carrying values. The approximate pollination value (i.e., multiplication of relative abundance, flower visitation rate, and pollen-carrying value) remained higher for some bees (e.g., *Apis cerana*, *Apis dorsata*, *Halictus acrocephalus*, *Nomia iridescens*, and *Tetragonula iridipennis*). Butterflies also provided significant pollination services to curry flowers, as diverse species visited flowers legitimately and had pollen content on their bodies. However, we are not able to estimate the pollen-carrying values of a few butterfly species, especially those whose abundances were low at the study sites. Most insect species are generalist visitors, and several research works are available regarding the pollination contribution of butterflies [34,35,36], honeybees [37,38], solitary bees [39,40] and stingless bees [20,41,42]. However, the report about diverse pollinator communities and spatial variation in floral visitors’ activity and their interactions with curry plants is the first in the current study.

## 5. Conclusions

Diverse insects (e.g., butterflies, flies, honeybees, moths, solitary bees, stingless bees, and wasps) visited curry flowers. Abundant floral visitors were *Apis cerana*, *Apis dorsata*, *Appias libythea*, *Halictus acrocephalus*, *Nomia iridescens*, and *Tetragonula iridipennis*. Floral visitors’ abundance, diversity, and richness varied from region to region (following vegetation cover types) and also daytime-wise. Comparatively higher abundance, richness, and diversity were recorded in dense vegetation areas than in medium- and low-density vegetation areas. From daytime-wise consideration, higher values were obtained during 10.00–14.00 h. The flower handling times were lower, and visitation rates were higher for most flower-visiting species in the dense vegetation zone than in the other zones. The proportions of nectar foragers and mixed foragers varied temporally, with higher percentages of mixed foragers recorded during the early morning (6.00–10.00 h). Regarding the approximate pollination value (combining relative abundance, visitation rate, and pollen carrying value), effective pollinators of curry were *Apis cerana*, *Apis dorsata*, *Appias libythea*, *Halictus acrocephalus*, *Nomia iridescens*, and *Tetragonula iridipennis*. However, zone-wise variation in effective pollinators was also recorded. The current study uncovered the visitor composition of curry, demonstrated spatial and temporal variations in visitors’ abundance, diversity, richness, and interactions, and will help conservation biology.

## Figures and Tables

**Table 1:** Floral visitors of curry plants in West Bengal, India.

Insect Order	Family	Insect Species
Diptera	Rhiniidae	*Stomorhina discolor* *^#^
	Stratiomyidae	*Oplodontha viridula*
Hymenoptera	Apidae	*Amegilla zonata*, *Apis cerana*, *Apis dorsata*, *Apis florea* ^∆^, *Ceratina binghami*, *Ceratina compacta*, *Tetragonula iridipennis*, *Thyreus nitidulus ^#^*^∆^
	Halictidae	*Halictus acrocephalus*, *Lasioglossum funebre*, *Nomia iridescens* ^∆^, *Sphecodes gibbus ^#^*^∆^
	Scoliidae	*Scolia soror* ^∆^
Lepidoptera	Erebidae	*Syntomoides imaon*
	Hesperiidae	*Ancistroides folus ^#^*^∆^, *Baoris farri*, *Suastus gremius*, and *Telicota colon*
	Lycaenidae	*Anthene lycaenina ^#^*^∆^, *Catochrysops strato* ^∆^, *Chilades lajus ^#^*^∆^, *Chilades pandava* ^∆^, *Jamides bochus ^#^*^∆^, *Rapala manea*, *Rapala varuna*, and *Tarucus indica ^#^*^∆^
	Nymphalidae	*Danaus chrysippus*, *Danaus genutia ^#^*^∆^, *Euploea core*, *Junonia almana ^#^*^∆^, *Junonia atlites*, *Junonia iphita*, *Mycalesis perseus ^#^*^∆^, *Tirumala limniace*
	Papilionidae	*Pachliopta hector*, *Papilio demoleus ^#^*^∆^, *Papilio polytes*,
	Pieridae	*Appias libythea*, *Catopsilia pomona*, *Eurema blanda*, *Eurema hecabe*, *Leptosia nina*, and *Pareronia hippie*

* absent in dense vegetation zone, ^#^ absent in medium-density vegetation zone, ^∆^ absent in low-density vegetation zone.

**Table 2:** Flower-visiting species richness (Margalef’s index *D* for sample wise) on curry plants (*Bergera koenigii*). Values are given as mean ± standard deviation. Different superscript letters after mean values (given in a single column for daytime slots in each zone and the last row for three zones) indicate significant differences (Dunn’s post hoc test at 5%).

Daytime	Values of *D*		
	Dense Vegetation	Medium-Density Vegetation	Low-Density Vegetation
6.00–8.00 h	0.35 ^c^ ± 0.64	0.33 ^c^ ± 0.60	0.21 ^b^ ± 0.48
8.00–10.00 h	1.06 ^b^ ± 0.63	0.86 ^ab^ ± 0.56	0.63 ^a^ ± 0.63
10.00–12.00 h	1.47 ^a^ ± 0.52	1.12 ^a^ ± 0.47	0.86 ^a^ ± 0.64
12.00–14.00 h	1.35 ^a^ ± 0.53	1.06 ^a^ ± 0.52	0.91 ^a^ ± 0.58
14.00–16.00 h	0.93 ^b^ ± 0.58	0.77 ^b^ ± 0.62	0.67 ^a^ ± 0.61
16.00–18.00 h	0.45 ^c^ ± 0.64	0.47 ^c^ ± 0.68	0.33 ^b^ ± 0.60
Overall	0.94 ^a^ ± 0.72	0.77 ^b^ ± 0.64	0.60 ^c^ ± 0.64

**Table 3:** Diversity of flower-visiting species (Shannon–Weiner index *H*’ for sample-wise) on curry plants (*Bergera koenigii*). Values are given as mean ± standard deviation. Different superscript letters after mean values (given in a single column for daytime slots in each zone and the last row for three zones) indicate significant differences (Dunn’s post hoc test at 5%).

Daytime	Values of *H*’		
	Dense Vegetation	Medium-Density Vegetation	Low-Density Vegetation
6.00–8.00 h	0.22 ^c^ ± 0.36	0.18 ^d^ ± 0.32	0.16 ^c^ ± 0.31
8.00–10.00 h	0.83 ^b^ ± 0.36	0.72 ^c^ ± 0.43	0.58 ^b^ ± 0.40
10.00–12.00 h	1.16 ^a^ ± 0.24	1.11 ^a^ ± 0.21	0.87 ^a^ ± 0.30
12.00–14.00 h	1.09 ^a^ ± 0.31	0.96 ^b^ ± 0.28	0.80 ^a^ ± 0.35
14.00–16.00 h	0.83 ^b^ ± 0.33	0.78 ^c^ ± 0.33	0.54 ^b^ ± 0.41
16.00–18.00 h	0.33 ^c^ ± 0.39	0.28 ^d^ ± 0.39	0.21 ^c^ ± 0.33
Overall	0.74 ^a^ ± 0.48	0.67 ^a^ ± 0.47	0.53 ^b^ ± 0.44

**Table 4:** Flower visitation rates (number of flowers visited per minute) and flower handling times of different floral visitors on curry plants (*Bergera koenigii*) in West Bengal.

Floral Visitors	Flower Visitation Rate			Flower Handling Time (s)		
	Dense Vegetation	Medium-Density Vegetation	Low-Density Vegetation	Dense Vegetation	Medium-Density Vegetation	Low-Density Vegetation
▪ Diptera						
*Oplodontha viridula*	-	-	-	-	-	-
*Stomorhina discolor*	-	-	-	-	-	-
▪ Hymenoptera						
*Amegilla zonata*	14.35 ± 3.47	14.20 ± 3.25	13.95 ± 3.03	1.42 ± 0.84	1.48 ± 0.85	1.54 ± 0.93
*Apis cerana*	11.05 ^a^ ± 2.49	9.90 ^b^ ± 2.40	9.12 ^c^ ± 2.43	3.66 ^c^ ± 1.15	4.03 ^b^ ± 1.24	4.32 ^a^ ± 1.41
*Apis dorsata*	19.53 ^a^ ± 3.68	16.10 ^b^ ± 4.43	14.98 ^c^ ± 3.81	1.39 ^c^ ± 0.59	1.59 ^b^ ± 0.51	1.81 ^a^ ± 0.54
*Apis florea*	12.07 ± 2.24	10.87 ± 2.33	-	3.43 ± 0.74	3.89 ± 0.96	-
*Ceratina binghami*	8.08 ^a^ ± 2.50	6.87 ^b^ ± 2.27	5.83 ^c^ ± 2.02	7.45 ± 3.87	8.07 ± 4.13	8.30 ± 4.32
*Ceratina compacta*	8.60 ^a^ ± 1.67	6.97 ^b^ ± 2.14	6.23 ^c^ ± 2.11	7.04 ^c^ ± 3.62	7.76 ^b^ ± 3.75	8.23 ^a^ ± 3.91
*Halictus acrocephalus*	8.92 ^a^ ± 2.49	6.82 ^b^ ± 2.27	5.35 ^c^ ± 2.15	6.22 ^c^ ± 2.50	7.85 ^b^ ± 3.33	9.55 ^a^ ± 3.67
*Lasioglossum funebre*	7.93 ^a^ ± 1.78	6.87 ^b^ ± 1.99	6.27 ^b^ ± 1.93	5.47 ^c^ ± 2.48	6.32 ^b^ ± 2.84	7.26 ^a^ ± 3.33
*Nomia iridescens*	11.92 ± 2.78	10.28 ± 2.90	-	5.44 ± 3.09	6.25 ± 3.69	-
*Scolia soror*	7.23 ± 1.94	7.07 ± 1.91	-	3.28 ± 0.83	3.72 ± 0.98	-
*Sphecodes gibbus*	11.22 ± 1.85	-	-	3.48 ± 0.86	-	-
*Tetragonula iridipennis*	2.95 ^a^ ± 1.03	2.30 ^b^ ± 0.98	1.88 ^c^ ± 0.80	11.76 ^c^ ± 3.71	14.03 ^b^ ± 4.04	16.34 ^a^ ± 4.40
*Thyreus nitidulus*	13.80 ± 3.27	-	-	3.17 ± 0.74	-	-
▪ Lepidoptera						
*Ancistroides folus*	3.02 ± 1.17	-	-	9.84 ± 6.12	-	-
*Anthene lycaenina*	2.15 ± 0.97	-	-	12.15 ± 8.37	-	-
*Appias libythea*	5.62 ^a^ ± 1.75	4.55 ^b^ ± 1.59	4.07 ^b^ ± 1.45	4.83 ^c^ ± 2.10	6.02 ^b^ ± 2.18	7.07 ^a^ ± 2.23
*Baoris farri*	3.25 ^a^ ± 1.10	3.00 ^b^ ± 1.02	2.83 ^c^ ± 0.95	7.83 ^c^ ± 5.36	9.75 ^b^ ± 5.92	11.47 ^a^ ± 5.98
*Catochrysops strato*	2.33 ± 1.06	2.03 ± 0.76	-	11.78 ± 8.07	13.37 ± 8.43	-
*Catopsilia pomona*	5.10 ^a^ ± 1.47	4.37 ^b^ ± 1.47	4.17 ^c^ ± 1.42	6.44 ^c^ ± 4.34	7.12 ^b^ ± 5.04	7.68 ^a^ ± 5.93
*Chilades lajus*	2.13 ± 0.97	-	-	11.85 ± 8.07	-	-
*Chilades pandava*	2.35 ± 0.99	2.03 ± 0.93	-	11.43 ± 7.84	13.57 ± 8.21	-
*Danaus chrysippus*	4.37 ± 1.61	4.10 ± 1.32	4.03 ± 1.27	5.39 ± 4.47	6.43 ± 5.04	7.88 ± 5.62
*Danaus genutia*	4.23 ± 1.68	-	-	5.12 ± 4.38	-	-
*Euploea core*	4.63 ^a^ ± 1.61	4.25 ^b^ ± 1.63	4.10 ^c^ ± 1.42	6.37 ^c^ ± 4.96	7.36 ^b^ ± 5.72	8.28 ^a^ ± 6.45
*Eurema blanda*	3.87 ^a^ ± 1.66	3.63 ^b^ ± 1.61	3.37 ^c^ ± 1.47	5.81 ^c^ ± 3.82	6.82 ^b^ ± 4.65	7.79 ^a^ ± 5.43
*Eurema hecabe*	4.12 ^a^ ± 1.63	3.83 ^b^ ± 1.51	3.53 ^c^ ± 1.28	5.46 ^c^ ± 3.65	6.54 ^b^ ± 4.39	7.42 ^a^ ± 5.22
*Jamides bochus*	2.23 ± 0.90	-	-	11.96 ± 8.22	-	-
*Junonia almana*	3.68 ± 1.42	-	-	5.38 ± 3.87	-	-
*Junonia atlites*	4.37 ^a^ ± 1.63	3.52 ^b^ ± 1.20	3.25 ^c^ ± 1.19	6.73 ^c^ ± 4.86	7.94 ^b^ ± 5.79	9.12 ^a^ ± 6.66
*Junonia iphita*	2.40 ± 1.07	2.13 ± 0.94	2.03 ± 0.96	11.04 ± 7.49	13.53 ± 7.72	15.16 ± 7.93
*Leptosia nina*	-	-	-	-	-	-
*Mycalesis perseus*	2.03 ± 0.96	-	-	10.83 ± 7.84	-	-
*Pachliopta hector*	6.53 ^a^ ± 2.30	5.90 ^b^ ± 1.58	5.47 ^c^ ± 1.59	4.71 ^c^ ± 2.28	5.34 ^b^ ± 2.57	5.82 ^a^ ± 2.81
*Papilio demoleus*	6.70 ± 1.95	-	-	4.78 ± 2.19	-	-
*Papilio polytes*	6.13 ± 1.87	5.93 ± 1.68	5.78 ± 1.65	4.95 ± 2.24	5.62 ± 2.43	6.18 ± 2.67
*Pareronia hippie*	4.17 ^a^ ± 1.66	3.20 ^b^ ± 1.13	2.70 ^c^ ± 1.12	6.02 ^c^ ± 4.62	7.28 ^b^ ± 5.53	8.40 ^a^ ± 6.44
*Rapala manea*	2.28 ^a^ ± 0.94	1.97 ^b^ ± 0.89	1.83 ^c^ ± 0.83	12.27 ^c^ ± 8.19	13.71 ^b^ ± 8.73	15.25 ^a^ ± 9.28
*Rapala varuna*	2.47 ^a^ ± 1.01	2.17 ^b^ ± 0.91	1.90 ^c^ ± 0.84	11.73 ^c^ ± 7.94	13.26 ^b^ ± 8.54	14.83 ^a^ ± 8.92
*Suastus gremius*	3.15 ^a^ ± 1.09	2.77 ^b^ ± 1.14	2.25 ^c^ ± 0.98	9.96 ^c^ ± 5.72	12.46 ^b^ ± 8.06	14.42 ^a^ ± 10.12
*Syntomoides imaon*	11.60 ± 2.55	11.73 ± 2.72	11.93 ± 2.80	2.46 ± 0.61	2.68 ± 0.68	2.93 ± 0.73
*Tarucus indica*	1.95 ± 0.85	-	-	12.28 ± 8.23	-	-
*Telicota colon*	3.30 ^a^ ± 1.12	2.80 ^b^ ± 1.21	2.47 ^c^ ± 1.11	7.86 ^c^ ± 4.72	9.91 ^b^ ± 5.77	12.28 ^a^ ± 7.50
*Tirumala limniace*	5.68 ^a^ ± 1.86	5.27 ^b^ ± 1.60	4.90 ^c^ ± 1.37	5.87 ^c^ ± 3.30	6.54 ^b^ ± 3.99	7.28 ^a^ ± 5.22

Values are given as the mean ± standard deviation. Different superscript letters after the mean values (row-wise to a parameter for each insect species) indicate significant differences (DMRT at 5%).

**Table 5:** Pollen-carrying value (PCV) and approximate pollination value (APV) of floral visitors on curry plants (*Bergera koenigii*).

Floral Visitors	Pollen-Carrying Value			APV		
	PCV1	PCV2	PCV	Dense Vegetation	Medium-Density Vegetation	Low-Density Vegetation
▪ Diptera						
*Oplodontha viridula*	-	-	-	-	-	-
*Stomorhina discolor*	-	-	-	-	-	-
▪ Hymenoptera						
*Amegilla zonata*	1	0.5	1.5	26.69	36.42	34.53
*Apis cerana*	1	1	2	46.63	56.23	159.24
*Apis dorsata*	1	0.5	1.5	163.47	72.21	118.42
*Apis florea*	0.5	0.5	1	22.45	15.44	-
*Ceratina binghami*	0.5	0.5	1	12.04	5.84	4.78
*Ceratina compacta*	0.5	0.5	1	13.85	16.87	15.39
*Halictus acrocephalus*	0.5	1	1.5	121.22	181.89	136.18
*Lasioglossum funebre*	0.5	0.5	1	23.63	16.63	16.55
*Nomia iridescens*	1	0	1	208.48	90.67	-
*Scolia soror*	-	-	-	-	-	-
*Sphecodes gibbus*	-	-	-	-	-	-
*Tetragonula iridipennis*	0.5	1	1.5	26.37	52.03	57.61
*Thyreus nitidulus*	0.5	0	0.5	6.83	-	-
▪ Lepidoptera						
*Ancistroides folus*	-	-	-	-	-	-
*Anthene lycaenina*	-	-	-	-	-	-
*Appias libythea*	0.5	0	0.5	48.46	15.22	14.75
*Baoris farri*	-	-	-	-	-	-
*Catochrysops strato*	-	-	-	-	-	-
*Catopsilia pomona*	0.5	0	0.5	10.43	13.68	14.07
*Chilades lajus*	-	-	-	-	-	-
*Chilades pandava*	-	-	-	-	-	-
*Danaus chrysippus*	0.5	0	0.5	4.35	5.82	4.65
*Danaus genutia*	-	-	-	-	-	-
*Euploea core*	0.5	0	0.5	3.73	4.23	4.39
*Eurema blanda*	0.5	0	0.5	2.40	3.10	2.22
*Eurema hecabe*	0.5	0	0.5	2.31	2.45	2.03
*Jamides bochus*	-	-	-	-	-	-
*Junonia almana*	-	-	-	-	-	-
*Junonia atlites*	0.5	0	0.5	4.06	4.51	4.55
*Junonia iphita*	-	-	-	-	-	-
*Leptosia nina*	-	-	-	-	-	-
*Mycalesis perseus*	-	-	-	-	-	-
*Pachliopta hector*	-	-	-	-	-	-
*Papilio demoleus*	-	-	-	-	-	-
*Papilio polytes*	-	-	-	-	-	-
*Pareronia hippie*	0.5	0	0.5	3.11	3.18	2.67
*Rapala manea*	-	-	-	-	-	-
*Rapala varuna*	-	-	-	-	-	-
*Suastus gremius*	0.5	0	0.5	3.32	3.74	2.97
*Syntomoides imaon*	0.5	0	0.5	3.60	10.03	13.78
*Tarucus indica*	-	-	-	-	-	-
*Telicota colon*	-	-	-	-	-	-
*Tirumala limniace*	-	-	-	-	-	-

## Data Availability

Data are contained within the article and Supplementary Materials.

## Supplementary Materials

The following supporting information can be downloaded at: https://www.mdpi.com/article/10.3390/insects15020083/s1, Table S1. Relative abundances of different floral visitors on *Bergera koenigii* in West Bengal; Table S2. Daytime-wise flower visitation rates of visitors on curry flowers; Table S3. Daytime-wise flower handling time of visitors on curry flowers; Table S4. Daytime-wise proportion of different resource-collecting task allocation categories on curry flowers.
