# Supplementary material for: Spatial and Temporal Variations in Richness, Diversity and Abundance of Floral Visitors of Curry Plants (Bergera koenigii L.): Insights on Plant-Pollinator Interactions
Source: Insects. 2024 Jan 24;15(2):83. doi: 10.3390/insects15020083 (PMC10889569; doi:10.3390/insects15020083)
Supplement: Supplementary file 1 [file insects-15-00083-s001.zip › Supplementary Table S1.pdf]

Supplementary Table S1. Table S1. Relative abundances of different floral visitors on *Murraya koenigii* in West Bengal.

Floral visitors	Relative abundance (%)		
	Dense vegetation	Medium-density vegetation	Low-density vegetation
▪ Diptera			
Oplodontha viridula	-	-	0.99
Stomorhina discolor	0.37	1.28	0.82
▪ Hymenoptera			
Amegilla zonata	1.24	1.71	1.65
Apis cerana	2.11	2.84	8.73
Apis dorsata	5.58	2.99	5.27
Apis florea	1.86	1.42	-
Ceratina binghami	1.49	0.85	0.82
Ceratina compacta	1.61	2.42	2.47
Halictus acrocephalus	9.06	17.78	16.97
Lasioglossum funebre	2.98	2.42	2.64
Nomia iridescens	17.49	8.82	-
Scolia soror	4.47	1.42	-
Sphecodes gibbus	1.12	-	-
Tetragonula iridipennis	5.96	15.08	20.43
Thyreus nitidulus	0.99	-	-
▪ Lepidoptera			
Ancistroides folus	0.50	-	-
Anthene lycaenina	0.25	-	-
Appias libythea	14.64	6.69	7.25
Baoris farri	0.37	1.28	1.98
Catochrysops strato	0.62	1.42	-
Catopsilia pomona	4.09	6.26	6.75
Chilades lajus	0.25	-	-
Chilades pandava	0.50	1.50	-
Danaus chrysippus	1.99	2.84	2.31
Danaus genutia	0.74	-	-
Euploea core	1.61	1.99	2.14
Eurema blanda	1.24	1.71	1.32
Eurema hecabe	1.12	1.28	1.15
Jamides bochus	0.25	-	-
Junonia almana	0.62	-	-
Junonia atlites	1.86	2.56	2.80
Junonia iphita	1.36	1.28	0.99
Leptosia nina	0.62	0.57	0.49
Mycalesis perseus	0.99	-	-
Pachliopta hector	0.87	1.14	0.82
Papilio demoleus	0.74	-	-
Papilio polytes	1.24	1.28	1.15
Pareronia hippie	1.49	1.99	1.98
Rapala manea	0.62	0.57	0.82
Rapala varuna	0.50	0.43	0.82
Suastus gremius	2.11	2.70	2.64
Syntomoides imaon	0.62	1.71	2.31
Tarucus indica	0.37	-	-
Telicota colon	0.50	0.43	0.33
Tirumala limniace	0.99	1.28	1.15
