# Supplementary material for: Spatial and Temporal Variations in Richness, Diversity and Abundance of Floral Visitors of Curry Plants (Bergera koenigii L.): Insights on Plant-Pollinator Interactions
Source: Insects. 2024 Jan 24;15(2):83. doi: 10.3390/insects15020083 (PMC10889569; doi:10.3390/insects15020083)
Supplement: Supplementary file 1 [file insects-15-00083-s001.zip › Supplementary Table S2.pdf]

Supplementary Table S2. Daytime-wise flower visitation rates of visitors on curry flowers.

Floral visitors	Daytime-wise (h) flower visitation rate					
	6.00-8.00	8.00-10.00	10.00-12.00	12.00-14.00	14.00-16.00	16.00-18.00
▪ Diptera						
Oplodontha viridula	-	-	-	-	-	-
Stomorhina discolor	-	-	-	-	-	-
▪ Hymenoptera						
Amegilla zonata	12.27 ± 2.86	14.50 ± 3.12	16.73 ± 3.21	16.23 ± 3.16	14.33 ± 2.08	13.93 ± 2.95
Apis cerana	7.73 ± 1.70	10.97 ± 2.06	11.73 ± 2.46	11.10 ± 2.59	10.07 ± 2.13	8.53 ± 1.72
Apis dorsata	13.37 ± 3.11	17.53 ± 3.61	20.57 ± 4.31	19.37 ± 4.20	16.50 ± 3.14	13.90 ± 2.92
Apis florea	9.55 ± 1.93	11.95 ± 2.04	13.50 ± 2.13	12.25 ± 2.09	11.30 ± 1.96	10.25 ± 1.95
Ceratina binghami	6.23 ± 1.50	7.87 ± 1.62	8.97 ± 1.71	6.73 ± 1.54	6.17 ± 1.48	5.63 ± 1.52
Ceratina compacta	5.97 ± 1.57	7.53 ± 1.65	8.77 ± 1.76	8.03 ± 1.69	7.10 ± 1.64	6.23 ± 1.62
Halictus acrocephalus	4.67 ± 2.02	7.70 ± 2.68	9.00 ± 2.36	8.03 ± 2.33	7.10 ± 2.43	5.67 ± 2.09
Lasioglossum funebre	5.37 ± 1.42	6.53 ± 1.58	8.30 ± 1.63	7.63 ± 1.59	7.27 ± 1.61	7.03 ± 1.63
Nomia iridescens	8.65 ± 2.11	12.15 ± 2.21	12.75 ± 2.97	12.35 ± 3.17	11.15 ± 2.41	9.55 ± 2.42
Scolia soror	6.15 ± 1.34	7.65 ± 1.48	8.65 ± 1.52	7.20 ± 1.38	6.80 ± 1.36	6.45 ± 1.37
Sphecodes gibbus	9.20 ± 1.85	11.30 ± 1.96	12.20 ± 2.09	12 ± 2.04	11.50 ± 2.06	11.10 ± 2.05
Tetragonula iridipennis	1.87 ± 0.86	2.47 ± 0.97	2.97 ± 1.16	2.70 ± 1.02	2.23 ± 1.01	2.03 ± 0.81
Thyreus nitidulus	12.50 ± 3.12	13.80 ± 3.15	15.30 ± 3.19	14.20 ± 3.17	13.80 ± 3.17	13.20 ± 3.16
▪ Lepidoptera						
Ancistroides folus	2.10 ± 0.94	2.90 ± 0.98	3.70 ± 1.15	3.50 ± 1.09	3.10 ± 1.06	2.80 ± 1.05
Anthene lycaenina	1.70 ± 0.82	2.20 ± 0.95	2.70 ± 0.97	2.30 ± 0.96	2.10 ± 0.97	1.90 ± 0.96
Appias libythea	3.67 ± 1.37	5.07 ± 1.48	6.07 ± 1.72	5.47 ± 1.59	4.63 ± 1.33	3.57 ± 1.33
Baoris farri	2.17 ± 0.95	3.03 ± 1.02	3.83 ± 1.26	3.67 ± 1.24	2.97 ± 1.13	2.50 ± 1.07
Catochrysops strato	1.70 ± 0.84	2.25 ± 0.96	2.80 ± 0.99	2.35 ± 0.97	2.10 ± 0.98	1.85 ± 0.97
Catopsilia pomona	3.87 ± 1.32	4.63 ± 1.46	5.20 ± 1.49	4.87 ± 1.48	4.50 ± 1.45	4.23 ± 1.42
Chilades lajus	1.70 ± 0.83	2.30 ± 0.95	2.60 ± 0.98	2.40 ± 0.96	2.00 ± 0.87	1.80 ± 0.85
Chilades pandava	1.85 ± 0.87	2.20 ± 0.94	2.70 ± 0.96	2.35 ± 0.96	2.10 ± 0.95	1.95 ± 0.96
Danaus chrysippus	3.47 ± 1.25	4.20 ± 1.42	4.90 ± 1.67	4.43 ± 1.51	4.13 ± 1.40	3.87 ± 1.38
Danaus genutia	3.70 ± 1.38	4.30 ± 1.43	4.80 ± 1.65	4.40 ± 1.50	4.20 ± 1.42	4.00 ± 1.41
Euploea core	3.87 ± 1.39	4.43 ± 1.52	4.93 ± 1.68	4.57 ± 1.57	4.27 ± 1.49	3.90 ± 1.44
Eurema blanda	3.30 ± 1.22	3.77 ± 1.29	4.03 ± 1.37	3.83 ± 1.35	3.50 ± 1.31	3.27 ± 1.23
Eurema hecabe	3.57 ± 1.26	3.90 ± 1.36	4.33 ± 1.39	4.07 ± 1.37	3.73 ± 1.28	3.40 ± 1.25
Jamides bochus	1.90 ± 0.89	2.30 ± 0.94	2.70 ± 0.97	2.40 ± 0.95	2.20 ± 0.91	1.90 ± 0.90
Junonia almana	3.20 ± 1.17	3.80 ± 1.47	4.30 ± 1.68	3.90 ± 1.59	3.60 ± 1.48	3.30 ± 1.26
Junonia atlites	3.40 ± 1.24	3.83 ± 1.36	4.17 ± 1.39	3.97 ± 1.37	3.60 ± 1.35	3.30 ± 1.32
Junonia iphita	1.87 ± 0.88	2.27 ± 0.95	2.60 ± 1.04	2.43 ± 0.99	2.13 ± 0.94	1.83 ± 0.87
Leptosia nina	-	-	-	-	-	-
Mycalesis perseus	1.70 ± 0.85	2.00 ± 0.89	2.50 ± 0.97	2.20 ± 0.94	2.00 ± 0.91	1.80 ± 0.89
Pachliopta hector	5.17 ± 2.12	5.80 ± 2.28	6.53 ± 2.42	6.43 ± 2.40	6.17 ± 2.26	5.73 ± 2.25
Papilio demoleus	6.00 ± 1.85	6.90 ± 1.96	7.50 ± 2.14	7.20 ± 2.07	6.50 ± 1.93	6.10 ± 1.91
Papilio polytes	5.13 ± 1.55	5.76 ± 1.62	6.50 ± 1.73	6.47 ± 1.71	6.13 ± 1.63	5.70 ± 1.62
Pareronia hippie	3.10 ± 1.45	3.43 ± 1.53	3.73 ± 1.64	3.60 ± 1.61	3.23 ± 1.51	3.07 ± 1.42
Rapala manea	1.67 ± 0.82	2.03 ± 0.94	2.47 ± 1.13	2.30 ± 1.10	1.97 ± 0.95	1.73 ± 0.80
Rapala varuna	1.77 ± 0.85	2.27 ± 0.92	2.63 ± 1.07	2.40 ± 1.02	2.13 ± 0.98	1.87 ± 0.93
Suastus gremius	2.23 ± 1.03	2.77 ± 1.12	3.13 ± 1.27	2.97 ± 1.22	2.70 ± 1.23	2.53 ± 1.08
Syntomoides imaon	9.67 ± 2.37	11.53 ± 2.54	13.23 ± 2.85	12.73 ± 2.79	12.07 ± 2.68	11.27 ± 2.53
Tarucus indica	1.40 ± 0.76	1.80 ± 0.82	2.40 ± 0.95	2.20 ± 0.91	2.00 ± 0.88	1.90 ± 0.87
Telicota colon	2.37 ± 1.01	2.93 ± 1.22	3.20 ± 1.34	3.17 ± 1.31	2.83 ± 1.17	2.67 ± 1.13
Tirumala limniace	4.83 ± 1.32	5.40 ± 1.51	5.83 ± 1.69	5.43 ± 1.53	5.23 ± 1.48	4.97 ± 1.37
