# Supplementary material for: Spatial and Temporal Variations in Richness, Diversity and Abundance of Floral Visitors of Curry Plants (Bergera koenigii L.): Insights on Plant-Pollinator Interactions
Source: Insects. 2024 Jan 24;15(2):83. doi: 10.3390/insects15020083 (PMC10889569; doi:10.3390/insects15020083)
Supplement: Supplementary file 1 [file insects-15-00083-s001.zip › Supplementary Table S3.pdf]

Supplementary Table S3. Daytime-wise flower handling time of visitors on curry flowers.

Floral visitors	Daytime-wise (h) flower handling time (sec)					
	6.00-8.00	8.00-10.00	10.00-12.00	12.00-14.00	14.00-16.00	16.00-18.00
▪ Diptera						
Oplodontha viridula	-	-	-	-	-	-
Stomorhina discolor	-	-	-	-	-	-
▪ Hymenoptera						
Amegilla zonata	1.76 ± 0.46	1.53 ± 0.42	1.38 ± 0.39	1.27 ± 0.38	1.39 ± 0.41	1.54 ± 0.43
Apis cerana	5.44 ± 1.69	4.52 ± 1.11	3.69 ± 0.90	3.23 ± 0.68	3.38 ± 0.65	3.77 ± 0.97
Apis dorsata	2.01 ± 0.54	1.67 ± 0.58	1.42 ± 0.48	1.32 ± 0.51	1.52 ± 0.55	1.65 ± 0.53
Apis florea	4.17 ± 1.42	3.86 ± 1.27	3.45 ± 1.15	3.23 ± 1.08	3.49 ± 1.32	3.74 ± 1.38
Ceratina binghami	9.13 ± 4.87	8.17 ± 4.33	7.06 ± 3.92	7.25 ± 4.11	7.71 ± 4.30	8.32 ± 4.35
Ceratina compacta	8.65 ± 4.36	7.52 ± 3.97	7.13 ± 3.61	7.28 ± 3.65	7.64 ± 3.83	7.86 ± 3.92
Halictus acrocephalus	10.30 ± 5.29	8.76 ± 3.34	7.53 ± 2.58	6.34 ± 2.85	6.81 ± 2.15	7.50 ± 2.15
Lasioglossum funebre	7.07 ± 3.24	6.21 ± 2.96	5.62 ± 2.63	5.92 ± 2.72	6.35 ± 2.98	6.93 ± 3.17
Nomia iridescens	7.70 ± 3.45	5.89 ± 3.03	5.40 ± 2.78	5.01 ± 2.01	5.29 ± 2.47	5.56 ± 1.88
Scolia soror	4.11 ± 1.40	3.64 ± 1.25	3.24 ± 1.04	3.06 ± 0.93	3.28 ± 1.07	3.67 ± 1.31
Sphecodes gibbus	4.06 ± 1.32	3.83 ± 1.26	3.37 ± 1.09	2.91 ± 0.84	3.16 ± 0.92	3.55 ± 1.16
Tetragonula iridipennis	16.16 ± 5.09	14.49 ± 4.77	12.66 ± 3.74	13.02 ± 4.42	13.83 ± 4.00	14.10 ± 4.03
Thyreus nitidulus	3.48 ± 1.16	3.19 ± 1.02	2.72 ± 0.76	2.89 ± 0.79	3.23 ± 1.04	3.51 ± 1.19
▪ Lepidoptera						
Ancistroides folus	11.03 ± 4.23	10.35 ± 3.94	8.97 ± 3.52	9.23 ± 3.67	9.54 ± 3.85	9.92 ± 3.91
Anthene lycaenina	14.46 ± 4.85	12.89 ± 4.37	10.82 ± 3.91	11.24 ± 4.19	11.56 ± 4.22	11.93 ± 4.36
Appias libythea	7.50 ± 2.49	6.06 ± 1.98	5.69 ± 2.34	5.08 ± 2.12	5.56 ± 2.10	5.97 ± 2.44
Baoris farri	10.85 ± 4.08	10.14 ± 3.95	8.83 ± 3.87	9.12 ± 3.89	9.42 ± 3.92	9.74 ± 4.12
Catochrysops strato	14.76 ± 4.97	13.22 ± 4.62	11.46 ± 4.39	11.65 ± 4.38	11.97 ± 4.43	12.39 ± 4.58
Catopsilia pomona	7.72 ± 3.68	7.08 ± 3.46	6.21 ± 3.32	6.73 ± 3.48	7.23 ± 3.57	7.51 ± 3.63
Chilades lajus	13.84 ± 5.72	12.65 ± 5.41	10.98 ± 5.13	10.81 ± 5.04	11.15 ± 5.16	11.67 ± 5.32
Chilades pandava	14.67 ± 6.13	13.12 ± 5.38	11.43 ± 5.26	11.58 ± 5.29	11.84 ± 5.32	12.36 ± 5.36
Danaus chrysippus	7.01 ± 2.31	6.33 ± 2.16	6.04 ± 1.95	6.38 ± 2.17	6.49 ± 2.23	7.15 ±2.38
Danaus genutia	5.60 ± 1.63	5.07 ± 1.42	4.56 ± 1.27	4.89 ± 1.35	5.12 ± 1.44	5.48 ± 1.57
Euploea core	7.94 ± 2.54	7.37 ± 2.35	6.62 ± 2.17	6.95 ± 2.24	7.41 ± 2.38	7.73 ± 2.45
Eurema blanda	7.17 ± 2.36	6.54 ± 2.17	6.15 ± 1.94	6.69 ± 2.19	6.83 ± 2.26	7.46 ± 2.42
Eurema hecabe	6.93 ± 2.25	6.28 ± 2.06	5.84 ± 1.83	6.28 ± 2.08	6.39 ± 2.17	7.12 ± 2.31
Jamides bochus	14.25 ± 6.37	12.63 ± 5.84	10.79 ± 5.31	11.02 ± 5.42	11.34 ± 5.52	11.73 ± 5.61
Junonia almana	5.82 ± 1.69	5.26 ± 1.47	4.83 ± 1.24	5.14 ± 1.37	5.52 ± 1.55	5.71 ± 1.62
Junonia atlites	8.32 ± 2.71	7.69 ± 2.42	7.25 ± 2.18	7.84 ± 2.59	7.93 ± 2.61	8.55 ± 2.74
Junonia iphita	15.66 ± 6.85	13.91 ± 6.24	12.27 ± 5.69	12.23 ± 5.65	12.65 ± 5.82	12.74 ± 5.93
Leptosia nina	-	-	-	-	-	-
Mycalesis perseus	11.97 ± 5.93	11.43 ± 5.41	10.14 ± 4.92	10.30 ± 5.13	10.32 ± 5.14	10.82 ± 5.37
Pachliopta hector	5.73 ± 1.72	5.25 ± 1.56	4.72 ± 1.37	5.03 ± 1.48	5.41 ± 1.63	5.60 ± 1.68
Papilio demoleus	5.23 ± 1.47	4.64 ± 1.28	4.28 ± 1.03	4.50 ± 1.24	4.86 ± 1.32	5.17 ± 1.39
Papilio polytes	5.96 ± 1.74	5.48 ± 1.56	5.05 ± 1.35	5.31 ± 1.48	5.83 ± 1.63	5.87 ± 1.66
Pareronia hippie	7.83 ± 2.49	7.26 ± 2.23	6.57 ± 2.06	6.84 ± 2.10	7.29 ± 2.25	7.61 ± 2.31
Rapala manea	16.12 ± 7.13	14.46 ± 6.75	12.73 ± 6.21	12.75 ± 6.26	13.16 ± 6.47	13.24 ±6.50
Rapala varuna	15.70 ± 6.95	13.82 ± 6.28	12.30 ± 5.63	12.29 ± 5.64	12.58 ± 5.71	12.95 ± 5.83
Suastus gremius	14.41 ± 6.38	12.94 ± 5.82	11.28 ± 5.39	11.35 ± 5.42	11.61 ± 5.53	12.09 ± 5.68
Syntomoides imaon	2.83 ± 0.62	2.62 ± 0.59	2.39 ± 0.53	2.68 ± 0.56	2.74 ± 0.58	2.88 ± 0.64
Tarucus indica	14.21 ± 6.27	12.84 ± 5.94	11.45 ± 5.52	11.47 ± 5.54	11.53 ± 5.59	12.18 ± 5.72
Telicota colon	11.14 ± 5.46	10.63 ± 5.27	9.21 ± 4.98	9.43 ± 5.03	9.62 ±5.12	10.07 ± 5.27
Tirumala limniace	7.05 ± 3.22	6.39 ± 3.05	5.94 ± 2.87	6.31 ± 2.91	6.82 ± 3.14	6.87 ± 3.16
