# Supplementary material for: Spatial and Temporal Variations in Richness, Diversity and Abundance of Floral Visitors of Curry Plants (Bergera koenigii L.): Insights on Plant-Pollinator Interactions
Source: Insects. 2024 Jan 24;15(2):83. doi: 10.3390/insects15020083 (PMC10889569; doi:10.3390/insects15020083)
Supplement: Supplementary file 1 [file insects-15-00083-s001.zip › Supplementary Table S4.pdf]

**Supplementary Table S4.** Daytime-wise proportion of different resource-collecting task allocation categories on curry flowers.

| Floral visitors | Daytime-wise (h) percentage of different categories | | | | | | |
|---|---|---|---|---|---|---|---|
| | 6.00-8.00 | 8.00-10.00 | 10.00-12.00 | 12.00-14.00 | 14.00-16.00 | 16.00-18.00 | Overall |
| **Floral visitors collected nectar and pollen grains** | | | | | | | |
| ▪ Diptera | | | | | | | |
| *Oplodontha viridula* | - | - | - | - | - | - | - |
| *Stomorhina discolor* | - | - | - | - | - | - | - |
| ▪ Hymenoptera | | | | | | | |
| *Amegilla zonata* | | | | | | | |
| Nectar foragers | 16.67 ± 6.17 | 20 ± 7.56 | 47.33 ± 9.61 | 69.33 ± 7.04 | 100 | 100 | 58.89 ± 34.69 |
| Pollen foragers | 0 | 0 | 0 | 0 | 0 | 0 | 0 |
| Mixed foragers | 83.33 ± 6.17 | 80 ± 7.56 | 52.67 ± 9.61 | 30.67 ± 7.04 | 0 | 0 | 41.11 ± 34.69 |
| *Apis cerana* | | | | | | | |
| Nectar foragers | 32 ± 11.46 | 38 ± 9.41 | 56 ± 9.86 | 72 ± 6.76 | 100 | 100 | 66.33 ± 28.22 |
| Pollen foragers | 0 | 0 | 0 | 0 | 0 | 0 | 0 |
| Mixed foragers | 68 ± 11.46 | 62 ± 9.41 | 44 ± 9.86 | 28 ± 6.76 | 0 | 0 | 33.67 ± 28.22 |
| *Apis dorsata* | | | | | | | |
| Nectar foragers | 70 ± 7.56 | 76 ± 9.86 | 82 ± 9.41 | 90.67 ± 7.04 | 100 | 100 | 86.44 ± 13.35 |
| Pollen foragers | 0 | 0 | 0 | 0 | 0 | 0 | 0 |
| Mixed foragers | 30 ± 7.56 | 24 ± 9.86 | 18 ± 9.41 | 9.33 ± 7.04 | 0 | 0 | 13.56 ± 13.35 |
| *Apis florea* | | | | | | | |
| Nectar foragers | 69 ± 11.01 | 73 ± 11.60 | 77 ± 9.49 | 88 ± 7.89 | 100 | 100 | 84.50 ± 14.78 |
| Pollen foragers | 0 | 0 | 0 | 0 | 0 | 0 | 0 |
| Mixed foragers | 31 ± 11.01 | 27 ± 11.60 | 23 ± 9.49 | 12 ± 7.89 | 0 | 0 | 15.50 ± 14.78 |
| *Ceratina binghami* | | | | | | | |
| Nectar foragers | 16.67 ± 9.58 | 21 ± 9.26 | 50 ± 9.93 | 72.33 ± 8.37 | 100 | 100 | 60 ± 36.62 |
| Pollen foragers | 0 | 0 | 0 | 0 | 0 | 0 | 0 |
| Mixed foragers | 83.33 ± 9.58 | 79 ± 9.26 | 50 ± 9.93 | 27.67 ± 8.37 | 0 | 0 | 40 ± 36.62 |
| *Ceratina compacta* | | | | | | | |
| Nectar foragers | 15 ± 9.74 | 18.67 ± 9.35 | 47.33 ± 10.26 | 70 ± 9.78 | 100 | 100 | 58.50 ± 37.84 |
| Pollen foragers | 0 | 0 | 0 | 0 | 0 | 0 | 0 |
| Mixed foragers | 85 ± 9.74 | 81.33 ± 9.35 | 52.67 ± 10.26 | 30 ± 9.78 | 0 | 0 | 41.50 ± 37.84 |
| *Halictus acrocephalus* | | | | | | | |
| Nectar foragers | 8 ± 7.75 | 12 ± 10.82 | 39.33 ± 9.61 | 64 ± 9.86 | 100 | 100 | 53.89 ± 38.44 |
| Pollen foragers | 0 | 0 | 0 | 0 | 0 | 0 | 0 |
| Mixed foragers | 92 ± 7.75 | 88 ± 10.82 | 60.67 ± 9.61 | 36 ± 9.86 | 0 | 0 | 46.11 ± 38.44 |
| *Lasioglossum funebre* | | | | | | | |
| Nectar foragers | 15.67 ± 9.26 | 19.33 ± 8.68 | 49 ± 8.27 | 71 ± 7.48 | 100 | 100 | 59.27 ± 36.91 |
| Pollen foragers | 0 | 0 | 0 | 0 | 0 | 0 | 0 |
| Mixed foragers | 84.33 ± 9.26 | 80.67 ± 8.68 | 51 ± 8.27 | 29 ± 7.48 | 0 | 0 | 40.83 ± 36.91 |
| *Tetragonula iridipennis* | | | | | | | |
| Nectar foragers | 9.33 ± 5.94 | 16.67 ± 6.17 | 39.33 ± 7.04 | 66 ± 9.86 | 100 | 100 | 55.22 ± 37.12 |
| Pollen foragers | 5.33 ± 7.43 | 5.33 ± 7.43 | 4.67 ± 6.40 | 0 | 0 | 0 | 2.56 ± 5.52 |
| Mixed foragers | 85.33 ± 9.90 | 78 ± 8.62 | 56 ± 11.21 | 34 ± 9.86 | 0 | 0 | 42.22 ± 35.15 |
| **Floral visitors collected only nectar** | | | | | | | |
| *Ancistroides folus, Anthene lycaenina, Appias libythea, Baoris farri, Catochrysops strato, Catopsilia Pomona, Chilades lajus, Chilades pandava, Danaus chrysippus, Danaus genutia, Euploea core, Eurema blanda, Eurema hecabe, Jamides bochus, Junonia almanac, Junonia atlites, Junonia iphita, Leptosia nina, Mycalesis perseus, Nomia iridescens, Pachliopta hector, Papilio demoleus, Papilio polytes, Pareronia hippie, Rapala manea, Rapala varuna, Scolia soror, Sphecodes gibbus, Suastus gremius, Syntomoides imaon, Tarucus indica, Telicota colon, Tirumala limniace, Thyreus nitidulus* | | | | | | | |
